# Hepatitis C Virus Genotypes and Association With Viral Load in Yazd, Central Province of Iran

**DOI:** 10.5812/hepatmon.11705

**Published:** 2014-03-03

**Authors:** Hossein Hadinedoushan, Hasan Salmanroghani, Mohammad Kazem Amirbaigy, Mohsen Akhondi-Meybodi

**Affiliations:** 1 Immunology Department, Faculty of Medicine, Shahid Sadoughi University of Medical Sciences, Yazd, IR Iran; 2 Gastrology Department, Faculty of Medicine, Shahid Sadoughi Hospital, Shahid Sadoughi University of Medical Sciences, Yazd, IR Iran

**Keywords:** Hepatitis C Virus, Genotyping Techniques, Real-Time Polymerase Chain Reaction

## Abstract

**Background:**

Hepatitis C virus (HCV) is a major cause of liver disease. Infection with HCV is a global public health problem. The virus is classified into 6 genotypes and more than 80 subtypes named as a, b, c, etc. HCV genotyping has been an important parameter for the treatment of HCV infection.

**Objectives:**

The main aim of this study was to estimate the prevalence of HCV genotypes in Yazd, central province of Iran. In addition, the study investigated whether there was any association between HCV load and genotypes.

**Patients and Methods:**

This descriptive cross-sectional study was performed on samples suspicious of HCV infection from March 2010 to June 2013. Peripheral blood sample was obtained and screened for anti-HCV antibodies using Enzyme-Linked Immunosorbent Assay (ELISA). Then sera of anti-HCV positive samples were analyzed using quantitative polymerase chain reaction method. Plasma samples were used to determine the HCV genotypes of 1a, 1b, 2, 3, and 4 in 191 infected patients.

**Results:**

One hundred fifty-two out of 191 (79.6%) samples were from male patients. The mean of the patients’ age was 40.7 ± 11.9 years (range 21-75 years old). Sixty- three (33%) patients were included in 31-40 years group. The mean number of HCV in infected patients was 2.92 × 10^6 ^± 1.85 × 10^6^ copies/mL (Min: 508; Max: 2.75 × 10^8^ copies/mL). HCV genotype 3 was the predominant genotype (50.3%) followed by subtypes 1a (38.7%) and 1b (6.8%). The distribution of other HCV genotypes showed genotype 2 in 1.6% and mixed genotypes in 2.6% of positive samples. Genotype 3 was predominant in all age groups except 21-30 years of age group. We were unable to find any significant difference between mean viral load of the patients infected with genotype 3 and those infected with genotype 1 (1a and 1b).

**Conclusions:**

Findings of the present study showed that HCV genotype 3 was the predominant genotype followed by the subtypes 1a and 1b in Yazd, central province of Iran. In addition, there was no difference between HCV load and genotypes 1 and 3. HCV genotyping is recommended in other provinces of Iran.

## 1. Background

Hepatitis C virus (HCV) was recognized in 1989. HCV infection is a global public health problem and has been considered as a major cause of chronic liver disease worldwide. The disease has affected around 200 million people, which form to 3% of the world population (1). Approximately 350000 people infected with HCV die every year. Therefore, mortality rate due to HCV infection is very high. It is estimated that HCV is ten times more infectious than human immunodeficiency virus (2). HCV infection is the number one indication for liver transplantation in the United States (3). No vaccine or immunotherapy is available and effectiveness and high cost of treatment limit the current available treatments of HCV infection.

There is insufficient information about overall and exact estimation of HCV infection in Iran. Nearly all of the researches on HCV infection are restricted to some cities in Iran and the studies have been conducted on the samples of the healthy blood donors. The prevalence of HCV infection among blood donors in Iran provinces and cities was estimated to be 0.5% (95% CI: 0.4-0.6%). The highest prevalence of HCV infection was in Kashan (1.09%), a city in Isfahan province, and the lowest prevalence was in South Khorasan province (0.03%)(4). HCV is a small, enveloped RNA virus and belongs to the family of Flaviviridae. The diameter of HCV virion is about 55-65 nm (5). HCV genome is composed of a single positive strand RNA molecule of approximately 9.6 kb (6). It encodes a poly protein about 3010 amino acids in a single continuous open reading frame flanked at the 5' and 3' ends by untranslated regions (UTR). This poly protein precursor is cleaved by proteases, giving rise to ten mature structural and nonstructural proteins. The open reading frame comprised of three structural (C, E1, E2) and four nonstructural genes (NS2, NS3, NS4 and NS5) (7). Determination of HCV genotype is important in order to determine the duration of treatment and estimate the response to treatment and prognosis.

Currently, HCV is classified into 6 major genotypes and more than 80 subtypes named as a, b, c, etc. The previously reported HCV genotypes of 7 through 9 have been re-classified as subtypes of genotype 6 (8). Sequence inconsistency ranges from 31% to 33% among genotypes and from 20% to 25% among subtypes (9). Genotypes 1 to 3 show a worldwide distribution, whereas genotypes 4 to 6 are more geographically restricted (10). Genotype 4 is found mainly in North Africa and the Middle East, especially in Egypt (11). Genotype 5 is restricted to South Africa (12) and genotype 6 is found in South East Asia (13). Different methods have been used for HCV genotyping by researchers.

## 2. Objectives

Few studies with different methods have been conducted in order to figure out distributions of HCV genotypes in Iran. The aim of this study was to determine various HCV genotypes in infected samples in Yazd, central province of Iran. In addition, the study investigated whether there was any association between HCV load and genotypes.

## 3. Patients and Methods

This descriptive cross-sectional study was performed on samples from patients suspected of HCV infection. Samples were referred to Bou-Ali Pathobiology Laboratory as the reference molecular laboratory in Yazd, Iran, from March 2010 to June 2013. A questionnaire was given to 191 patients who had positive results for HCV test. The questionnaire included variables such as age and gender. The research protocol was approved by Ethic Committee of Shahid Sadoughi University of Medical Sciences, Yazd, Iran. A written consent form was signed by the participants. Five milliliter of each peripheral blood sample was taken and screened for anti-HCV antibodies using a commercial Enzyme-Linked Immunosorbent Assay (ELISA) kit (Diapro, Diaplus, Italy). Then, sera of the samples with positive results for anti-HCV were analyzed using real-time polymerase chain reaction (RT-PCR) method.

### 3.1. HCV Viral Load Assessment

The viral load in serum samples were determined accordance to the manufacturer instruction of the kit (AjRoboscreen GmbH, Germany). The Rotor-Gene 6000 (Corbett Research, Sydney, Australia) was used to determine the viral load. Analytical sensitivity of the kit was 200 copies/ml.

Plasma samples were used to determine the genotypes of HCV in infected patients. We used RT-PCR method for determining the HCV genotypes. This method is an in vitro nucleic acid amplification test for determination of HCV-RNA genotype by reverse transcription and nucleic acid amplification using HCV genotype specific primers. It is able to recognize HCV genotypes 1a, 1b, 2, 3, and 4. The procedure of the test is described briefly below.

### 3.2. Extraction of HCV RNA From Plasma

For extraction of HCV RNA, the procedure was done in accordance with the kit instruction (Sacace Biotechnologies S.r.l, Italy). Briefly, 150 µL of plasma sample and 600 µL buffer (Sacace Biotechnologies S.r.l, Italy) containing carrier RNA were mixed in micro centrifuge tube and were incubated for 5 min at 70ºC and then 600 µL ethanol was added to the tube. Sample was loaded to Ribovirus column and centrifuged for 1 min. at 8000 xg. Thereafter, 500 µL buffer RAW was added to column and centrifuged again. Column was washed by 600 µL buffer RAV3 and finally, virus RNA was eluted by 50 µL buffer RE. Then, complementary DNA (cDNA) synthesis and RT-PCR for determining of HCV genotypes were done according to the instruction of the kit (Sacace Biotechnologies S.r.l, Italy).

Tubes were transferred into the Rotor-Gene 6000 (Corbett Research, Sydney, Australia). The PCR cycling conditions was as follows: 15 minutes at 95ºC, followed by 45 cycles of 20 seconds at 95ºC, and 40 seconds at 60ºC.

According to manufacturer's instruction (Sacace Biotechnologies S.r.l, Italy), the analytical sensitivity of the HCV Real-TM genotype kit was 100 Iu/mL. The diagnostic specificity and sensitivity of the tested kit were both 100%.

### 3.3. Statistical Analysis

The results were analyzed by SPSS v.16.0 software (SPSS Inc, Chicago, IL, USA). The results related to the age were shown as Mean ± SD and the copy of viruses in each milliliter of the blood were presented as Mean ± SEM. Mann-Whitney U test was used for determining any difference between HCV load and genotypes 1 (1a and 1b) and 3. P value of < 0.05 was considered statistically significant.

## 4. Results

One hundred and fifty-two (79.6%) out of 191 participants who were investigated with respect to HCV genotype, were males. The mean age of the subjects was 40.7 ± 11.9 years old (min: 21 and max: 75 years old). Moreover, the mode was 34-year-old. The mean age of the female patients was insignificantly higher than that of the male patients (43.6± 13.5 and 39.9 ± 11.5, respectively; P = 0.1). The mean HCV viral load in infected patients were 2.92×10^6 ^± 1.85×10^6^copies/mL (Min: 508 and Max: 2.75×10^8^ copies/mL). There was not any correlation between age and plasma viral load in male (R = 0.09, P = 0.3) and female patients (R = 0.04, P = 0.7). In addition, there was no significant difference between plasma viral load and sex (male: 3.4×10^6 ^± 2.3×10^6^; female: 9.9×10^5 ^± 5.1×10^5^; P = 0.3). Genotype 3 was detected in 96 (50.3%) samples which can be considered as the dominant type in our study. Subtypes 1a and 1b were detected in 74 (38.7%) and 13 (6.8%) samples, respectively. The results are shown in Table 1. Subjects were categorized into different age groups as follows: 21-30 years, 31-40 years, 41-50 years, and above 50 years. Sixty-three (33%) samples were obtained from the group age 31-40. Genotype 3 was the predominant one in all age groups except for 21-30 age group in which genotype 1a was predominant. The results are summarized in Table 2. There was no significant difference between average viral load of the patients infected with genotype 3 and genotype 1 (1a and 1b) (P = 0.15). Frequency distribution of HCV genotypes 3 and 1 (1a and 1b) among males and females is shown in Table 3. There was not any significant difference between genotypes 1 (1a and 1b) and 3 regarding gender.

**Table 1.** Distribution Frequency of HCV Genotypes and Plasma Viral Load in Province of Yazd ^a^

Genotype	Count	Plasma Viral Load
**3**	96 (50.3)	6.5×10^5 ^± 3.2×10^5^
**1a**	74 (38.7)	7.1×10^6 ^± 5.1×10^6^
**1b**	13 (6.8)	3.9×10^5 ^± 1.6×10^5^
**2**	3 (1.6)	2.8×10^4 ^± 1.3×10^4^
**1a and 3**	1 (0.5)	4.3×10^5 ^± 4.1×10^5^
**1a and 1b**	4 (2.1)	2.6×10^5 ^± 1.5×10^5^
**Total**	191 (100)	

^a^ Data are presented as No. (%) and mean ± SEM.

**Table 2.** Frequency Distribution of HCV Genotypes in Different Age Groups ^a^

Age groups	3	1a	1b	2	1a and 3	1a and 1b	Total
**21-30**	14 (7.5)	21 (11)	1 (0.5)	0 (0)	0 (0)	1 (0.5)	37 (19.5)
**31-40**	35 (18.5)	20 (10.5)	4 (2)	2 (1)	0 (0)	2 (1)	63 (33)
**41-50**	21 (11)	15 (8)	5 (2.5)	1 (0.5)	1 (0.5)	0 (0)	43 (22.5)
**> 50**	26 (13.5)	18 (9.5)	3 (1.5)	0 (0)	0 (0)	1 (0.5)	48 (25)
**Total**	96 (50.5)	74 (39)	13 (6.5)	3 (1.5)	1 (0.5)	4 (2)	191 (100)

^a^ Data are presented as No. (%).

**Table 3.** HCV Genotype and Plasma Viral Load Among Male and Female ^a^

	HCV Genotype		Total
	3	1 (1a and 1b)	
**Male**			
Count	77 (42)	70 (38)	147 (80)
Viral load, copies/mL	6.9×10^5 ^± 4.1×10^5^	5.8×10^6 ^± 4.4×10^6^	
**Female**			
Count	19 (10.5)	17 (9.5)	36 (20)
Viral load, copies/mL	6.1×10^5 ^± 2.3×10^5^	1.7×10^6 ^± 1.1×10^6^	
**Total**	96 (52.5)	87 (47.5)	183 (100)

^a^ Data are presented as No. (%) and mean ± SEM.

## 5. Discussion

In this research, we measured anti-HCV antibodies in samples taken from patients suspected of HCV infection and then the quantitative RT-PCR method was used to confirm HCV infection. Real-time techniques are known as the method of choice for HCV infection diagnose and monitoring in European and American Liver Society guidelines (14, 15).Samples with positive results were used to define the genotypes of HCV. HCV genotyping has been an important parameter to determine both the likelihood of response to therapy and duration of therapy needed (16, 17). Genotypes 1 and 4 are more resistant to combination therapy with interferon and ribavirin than genotypes 2 and 3 (18).

To our knowledge, it was the first study on the HCV genotyping among people infected with HCV in Yazd, central province of Iran. RT-PCR method was used for determining HCV genotypes. The kit used in this research was able to determine HCV genotypes 1a, 1b, 2, 3 and 4. All samples' genotypes were identified in this study. Genotypes 1, 2, and 3 were found in infected samples that meant other HCV genotypes such as 4, 5, and 6 were not common in Yazd.

Findings of the present study showed that HCV genotype 3 was the predominant genotype (50.3%), followed by subtypes 1a (38.7%) and 1b (6.8%). These results were in agreed with results reported by Zarkesh-Esfahani et al. from Esfahan, neighboring province of Yazd. Their findings showed that predominant genotypes were 3a (61.2%), 1a (29.5%), and 1b (5.1%), consecutively (19). Neighboring provinces of Yazd and Esfahan can justify the consistent results. Results of the present study were different from results of a study of patients with chronic HCV infection in Tehran from March 2003 to December 2011 which demonstrated that subtype 1a was the most common subtype (44.9%) followed by subtype 3a (39.6%) and subtype 1b (11.3%). Subtype 3a was the most frequent genotype in patients under 40-year-old (41.5%) and subtype 1a was the most common genotype in subjects over 40-year-old (46.1%) (20).

The predominance of HCV genotype 3 in infected patients of Yazd was in agreement with some reports on genotyping of HCV isolates in different Asian countries such as Pakistan (21) and India (22). Sobia Attuallah et al. reported that HCV infection in patients with hepatitis in Pakistan was predominantly attributed to viral genotype 3 with the frequency rate of 78.9% (21). Massive immigration rates from Afghanistan and Pakistan, and traffic from these countries might have affected the distribution frequency of HCV genotypes in Yazd.

The pattern of HCV genotypes in this study was different from Persian Gulf countries such as Kuwait (23), United Arab Emirates (24), and Saudi Arabia, where genotype 4 was found in 74.2% of patients infected with HCV (25). We were unable to determine the HCV genotype 4 in our samples. The difference in race, routes of transmission, and socioeconomic factors might explain this discrepancy. Morice Y et al. reported that genotypes 3a and 1a were more frequent in patients infected with HCV through intravenous drug abuse (26). We are not sure whether our studied patients used poorly sterilized needles and syringes. High frequency of the HCV genotype 3 in patients infected in Yazd provides a sound hope for treatment as well as control of HCV infection. Genotype 3 requires shorter duration of treatment compared with other genotypes, with reduced associated costs and side effects. In the present study, genotype 1 was the second high prevalent genotype. Genotype 1 is prevalent in Europe, Canada (27), North and South America, and Australia (28). In accordance with this study, genotype 3 was the most common genotype in India followed by genotype 1 (22). Sixty-three patients were in 31-40 years of age group. It would be interesting to find out the factors involved in HCV infection in this age group. Genotype 1a was predominant in age group 21-30 while genotype 3 was predominant in other age groups which might be due to different routes of infection transmission in different age groups.

We were not able to find any significant difference between mean viral load level of the patients infected with genotype 3 and those infected with genotype 1 (1a and 1b). A higher viral load was expected in genotype 1 than other genotypes due to its more efficient replication. Results of the present study differed from those reported by Chakravarti A et al. showing that the mean viral load in patients infected with HCV genotype 1 was higher than those infected with genotypes 2 and 3 (22). In addition, no difference was found between HCV genotypes 3 and 1 regarding gender. Our results confirmed other same reports (29, 30). In summary, the present study highlighted that HCV genotype 3 was the predominant genotype in Yazd, central province of Iran followed by subtypes 1a and 1b. There was not any significant difference between the mean viral load in patients infected with HCV genotypes 1 and 3.
